# Once More, the Contact Point*Read before the Odontological Society of Chicago, January 7th, 1913. Reprinted from the April 1913 Dental Review.

**Published:** 1913-04-15

**Authors:** C. N. Johnson

**Affiliations:** Chicago, Ills.


### ONCE MORE, THE CONTACT POINT.*

BY C. N. JOHNSON, M.A., L.D.S., D.D.S., CHICAGO, ILLS.

As the title of this paper would imply, this is a reconsideration of a very old subject, and my only excuse for intruding it upon your notice at this time is the fact that so many men in the profession are continuing to ignore it, even after all that has been said and written upon it. The

*Read before the Odontological Society of Chicago, January 7th, 1913. Reprinted from the April 1913 DENTAL REVIEW.

reason for this is very hard to find because of all the factors involved in the filling of cavities in the proximal surfaces of the teeth this one of proper contact is the most important, with one exception. The chief consideration, of course, is to stop the decay, but aside from this the maintenance of proper contact becomes paramount. Without this there is little comfort to the patient and no assurance that the tooth operated on will be saved. Faulty contact leads inevitably to the lodgement of fibrous food between the teeth in the interproximal spaces, and this just as inevitably leads to destruction of the gum tissue occupying the space. If it stopped at this it would be serious enough, but unfortunately it goes further and does greater damage. It has frequently been pointed out that disastrous results follow the impaction of food between teeth causing injury to the tissues both soft and hard, and finally resulting in loss of the teeth. It probably never enters the mind of the average operator, even among those who have been impressed with the importance of maintaining the normal contact between teeth, that there was grave danger of losing a tooth as the direct result of bad contact, but the more this matter is studied the more certain it becomes that the loss of very many teeth may be traced directly to this cause. The reason this fact is not more universally recognized is because so much time usually elapses between the insertion of the faulty filling and the ultimate loss of the tooth. On account of this the filling is never suspected, but the blame is laid to some more or less obscure cause which results in recession of the gum and loosening of the tooth. There are, of course, various causes for gum recession, but it is safe to say that in these cases where such disastrous pockets are formed between teeth the initial lesion was started by the impaction of food due to faulty contact. Why cannot the profession be more observant of these cases and detect the original trouble in time to remedy it and save the tooth? Or better, why cannot they understand the philosophy of contact sufficiently to give fillings the correct form? Leaky contacts are usually manifest at a very early stage on examining any mouth. If fibers of food are found lodging between the proximal surfaces something is wrong and it should at once be remedied. Frequently the patient tolerates the annoyance because he does not quite know what the difficulty is and is not aware that there is a remedy. Sometimes he does not even mention it to the dentist till the dentist has called his attention to it. Then he is usually very alert to the situation and anxious to have something done, because of all the petty annoyances connected with the mastication of food this one of leaky contacts is the worst. And when it is known how serious the results may be there should be the heartiest co-operation between the practitioner and the patient to remedy the evil.

Fortunately for the safety of many teeth our patients are becoming more and more sensitive to the irritation of food in the interproximal spaces and will often at once make complaint when this trouble begins, and I am frank to confess that it sometimes taxes the ingenuity of the operator to overcome the difficulty. But no amount of effort should be considered too great to be expended upon the problem.

And this problem involves two considerations, each one of which I wish to discuss separately. One is faulty form of the contact point on the tooth affected, and the other faulty form of the occluding tooth on the opposite jaw. It was too much akin to carrying coals to Newcastle to deal at length before this society with the normal form of the contact point on the proximal surfaces of the human teeth. This has been drilled into the men of this section till they are as familiar with it as they are with the ten commandments —possibly some of them even more so—but a re-emphasis of a few points may be profitable to us all. It goes without saying that if fibrous food is not to be retained between the teeth the area of actual contact must be exceedingly small and that the form of the contract point shall be somewhat sharply rounded. I am aware that this does not seem to convey to the average observer an accurate idea of the actual form to be found on the proximal surfaces of normal human teeth, particularly the molars. The bucco-lingual width of these teeth would give the impression that the contact on the proximal surfaces must be more or less flattened and this impression is heightened by the ordinary method of examining them. If we look into a mouth with a full complement of teeth we see these broad molars apparently in contact for some distance bucco-lingually, but this is really not the case in all normal conditions. The deceptive thing about it is that when we look into a mouth under these conditions the interproximal spaces are filled either with gum tissue or foreign matter or saliva. This obscures the contact point and makes it appear much broader than it actually is. The only accurate way to learn the real area of contact between these teeth is to take two extracted human molars and place them side by side in the same relation which they occupied to each other in the mouth and then hold them up to the light. The area in which vision is obscured by the contact will give the actual area of that contact, and when this experiment is tried it will surprise most of us. It is soon found that even on teeth so broad as molars the area in actual contact is almost infinitesimal.

Of course it must be understood that I am speaking of normal contact. There are many cases of broad contact caused by the wearing of facets in the proximal surfaces of the teeth through the individual movement of the teeth one against the other, but this is clearly an abnormality and should not be considered in this connection, except as it teaches us a most vivid lesson. If the human enamel may be worn to the extent we often see it, then it is apparent that the hardest of our filling materials is none too hard to withstand this wear, and the lesson is that we shall make the contact points on our fillings as dense and hard as we can.

And in recent years I never approach this subject of small, narrow, rounded and hard contacts that I am not thankful for the gold inlay. By this method we are able to more easily and with less inconvenience to the patient make contacts that are true to nature's plan, than we can by the same form of fillings built in teeth in the mouth. Not that our master operators cannot make perfect contacts on foil and amalgam fillings. This has been demonstrated too often to call for argument, but that the fact is apparent that the rank and file of operators with the rank and file of patients will with greater ease and more certainty obtain good contacts with inlays than with fillings.

In this connection I wish to call attention to another matter relative to our operations on these cavities in the proximal surfaces and to beg your most careful consideration of it. This relates not only to the form and area of the contact point, but to the mesio-distal width of the filling or inlay. Usually when a tooth decays sufficiently to involve the incontact point the teeth drop together and this affects not only the two teeth immediately concerned, but it causes a loosening up of the contacts on several adjacent teeth. To "snug up" the arch on that side again as it should be involves not only the wedging apart of the affected teeth, but, through this means, a tightening of the adjacent teeth. Then the filling or inlay should be given such a form that this tight contact between all of the teeth shall be maintained and in order to do this it is necessary to make it the full mesio-distal width of the original tooth and in cases where the arch has been much loosened this width should be exaggerated. I look upon this tightening up of the arch as of the utmost importance. It gives a stability to the entire side of the jaw, which usually has been lacking ever since the original contact point broke down. It is in this one thing that I have had the very greatest satisfaction in the use of inlays. I invariably make them slightly wider mesio-distally than the wax model would indicate was necessary and then they are driven to place with a mallet blow. This will usually cause a temporary discomfort to the patient and the common remark is that the teeth feel too crowded. But this invariably passes away and leaves the integrity of the arch better maintained than if the inlay slipped to place easily. One thing in this connection must not be overlooked. In changing the position of the teeth in this way the occlusal relations are sometimes slightly interfered with to the extent of making a cusp impinge too hard against the opposing tooth. This may bring about a wrenching of the tooth and develop a soreness on closure. It may be necessary under these conditions to grind the offending cusp slightly to relieve the undue stress. In all of our operations this factor of normal occlusion must not be ignored.

Which brings me to the second consideration connected with the problem of maintaining comfortable mastication of fibrous foods, viz., the form of the occluding tooth. It will be found that in certain cases patients will complain of food packing between the teeth, even when the contact seems normal. This happens at times when the troublesome teeth have never developed decay and where the contacts seem satisfactory. It also occurs occasionally after we have done our utmost in the way of normal restoration of carious teeth and unless we understand the cause it is not only uncomfortable to the patient but very disconcerting to the operator. The fault is usually with the form of the opposing tooth. The sharp point of a cusp impinges between the affected teeth in such a way as to spring them apart on closure of the jaws and carry fibrous particles into the interproximal space. The remedy is extremely simple and usually brings almost instant relief, though occasionally in cases where the food has been lodging for some time it requires a reasonable period for the teeth to become firm enough not to spring apart. Ordinarily a bite in modeling compound should be taken and this poured in plaster. This will show the precise relation of the offending cusp to the affected teeth, and indicate where the cusp should be ground to remedy the difficulty. No hesitation should be had in grinding a cusp under these conditions and change it from a sharp wedge shape to a blunt grinding form which will not spring the teeth apart on closure of the jaws.

I had an experience in my own mouth which may be worth relating as indicative of the utter stupidity of a man when it comes to looking after his own comfort. Some years ago I began to have trouble with food packing between my left lower bicuspids. The teeth were not decayed and the contact seemed good. I examined the teeth from their buccal aspect and the occlusion appeared to be satisfactory, and yet the trouble continued. Finally I took a bite and made models. I saw at a glance the trouble. The lingual cusp of the upper bicuspid was turned in such a way as to constitute a very effective wedge to spring the lower bicuspids apart. I ground off this cusp and, presto! I was comfortable. Within the past year or two I have been experiencing great annoyance with food lodging between my second and third molars on each side. I attributed it to faulty natural form of the upper teeth, and as they were sound, I hesitated to have them cut into to remedy the trouble. The annoyance became so great and the interproximal spaces so uncomfortable that I began to fear pyorrhea pockets and was contemplating the extraction of the third molars as a means of safeguarding the second molars when it entered my idiotic head to take impressions of the parts and study the models. With ordinary intelligence and in view of my former experience this should have been done long ago, and it would have saved me much needless suffering. I found that the cusps on my lower molars had worn into sharp wedges and here was my difficulty. I may record that the grinding of these cusps was not performed with the same delicate care that I otherwise would have done the work, when each moment I realized what an abject imbecile I had been not to have thought of this before.

This entire subject of keeping our patients comfortable for mastication so that this function may be performed in its fullest efficiency is worthy of our most careful consideration as practitioners, and no matter how perfect our work may be otherwise, with margins above reproach, with density satisfactory and anchorage adequate, we have utterly failed in our best service to the patient if we have ignored this fundamental factor of proper contact. This is my apology for once more bringing the subject before you for consideration and discussion.

